# Random mutagenesis of super Koji (*Aspergillus oryzae*): improvement in production and thermal stability of α-amylases for maltose syrup production

**DOI:** 10.1186/s12866-018-1345-y

**Published:** 2018-11-28

**Authors:** Bushra Aleem, Muhammad Hamid Rashid, Neelam Zeb, Anam Saqib, Ayesha Ihsan, Mazhar Iqbal, Hazrat Ali

**Affiliations:** 1 National Institute for Biotechnology and Genetic Engineering (NIBGE), Jhang Road, P. O. Box 577, Faisalabad, Pakistan; 2 Pakistan Institute of Engineering and Applied Sciences (PIEAS), Nilore, Islamaabd Pakistan

**Keywords:** Strain development, Gamma rays, 2-deoxy D-glucose, Thermostability, Growth kinetics

## Abstract

**Background:**

Alpha-amylases hydrolyze 1,4 α-glycosidic bonds of starch and produce malto-oligosaccharides. It is an important enzyme generally applied in textile, food and brewing industries. Enhancement in thermal stability and productivity of enzymes are the two most sought after properties for industrial use. The *Aspergillus oryzae* (Koji) has Generally Recognized as Safe (GRAS) status and safe for use in food industry. Hence, Koji strain’s development for the screening of potent mutants, hyper producer of thermostable α-amylases, with desired attributes is the need of the time.

**Results:**

A process has been developed to improve super Koji (*A. oryzae* cmc1) strain through γ-rays treatment. The doses i.e. 0.60, 0.80, 1.00, 1.20 & 1.40 KGy gave more than 3.0 log kill. Initially, 52 Koji mutants resistant to 1% (*w/v*) Triton X-100 were selected. 2^nd^ screening was based on α-amylases hyper production and 23 mutants were sorted out by measuring clearing zones index (CI). Afterwards nine potent mutants, resistant to 2-deoxy D-glucose, were screened based on CI. These were further analyzed for thermal stability and productivity of α-amylase under submerged conditions. The mutants’ M-80(10), M-100(6) & M-120(5) gave about four fold increases in α-amylases productivity. The half life of M-100(6) α-amylase at 55 °C was 52 min and was highest among the mutants. Liquid Chromatography-Mass Spectrometry (LC-MS) analysis confirmed that mutants did not produce aflatoxins. Field Emission Scanning Electron Microscopy (FESEM) of Koji mycelia depicted that exposure to gamma rays increased rigidity of the mycelium. The potent Koji mutant M-100(6) was grown on soluble starch in 10L fermenter and produced 40.0 IU ml^-1^ of α-amylases with specific activity of 2461 IU mg^-1^ protein. Growth kinetic parameters were: *μ* = Specific growth rate= 0.069 h^-1^, t_d_ = Biomass doubling time= 10.0 h, *Y*_*p*/x_ = Product yield coefficient with respect to cell mass = 482 U g^-1^; *q*_p_= Specific rate of product formation= 33.29 U g^-1^ h^-1^.

**Conclusion:**

It was concluded that the developed five step screening process has great potential to generate potent mutants for the hyper production of thermostable enzymes through γ-rays mediated physical mutagenesis. The developed thermostable α-amylases of super Koji mutantM-100(6) has immense potential for application in saccharification process for maltose syrup production. Moreover, the developed five step strain’s development process may be used for the simultaneous improvement in productivity and thermal stability of other microbial enzymes.

**Electronic supplementary material:**

The online version of this article (10.1186/s12866-018-1345-y) contains supplementary material, which is available to authorized users.

## Background

Enzymes are the biocatalysts employed in catalyzing complex chemical reactions in biological systems. In the absence of enzymes, the reactions would have been very slow [1]. In the recent years a lot of studies are conducted for the use of enzymes in industry [2]. Scientific research on the use of industrial enzymes has opened new horizons to a lot of different fields including food production, brewing, pharmaceutical, medicine, textile and detergent as well as in research development. Biocatalysts have surpassed the inorganic catalysts as they are highly specific and exhibit high catalytic efficiency. Other important characteristic include no toxicity, water solubility, biodegradability, and mild operational conditions of pH, temperature, pressure [3].

The amylases (α-amylases, β-amylases, and glucoamylases) are one of the very useful families of enzymes in biotechnology and have widest range of industrial applications [4]. Alpha amylases (E.C. 3.2.1.1., 1,4-α-D-glucan glucanohydrolase) have a tendency to catalyze the hydrolysis of internal α-1, 4 glycosic linkage in starch, amylopectin and amylose converting them in maltose and glucose. These enzymes are critically important especially in the detergent and the food industries [5].

Many living beings are exploited for the production of amylases. The chemical hydrolysis in starch industries is replaced by microbial amylases. One of the biggest reasons for the use of microbial amylases is that they can be cultured in a controlled environment and the purification process is comparatively easy [6]. The use of enzymes from microbial source are preferred over conventional methods due to being environment friendly, better efficiency, and better quality products [7]. *A. oryzae* has been brought in use for many industrial processes since ancient time [8]. It is extensively used in the industry for the production of enzymes likewise amylases, proteases, lipases and many other secondary metabolites [9]. *A. oryzae* has been industries’ favorite because of its unique attributes including its ubiquitous nature, non-particular nutritional requirements and increased production of alpha amylase [10, 11].

Up until now many isolates of *A. oryzae* are put in use for plenty of industrial procedures for the production of organic acids, fermented food and enzymes [12]. To meet the increasing demand of α-amylase in industry, focus has shifted upon increasing enzyme production and finding new fast processes [13]. Detergent and food industries need enzymes that do not lose their activity after exposure to high process or operating temperatures. This has gathered significant attention [5]. Alpha amylases with increased thermal stability have acquired a pivotal position in many industries such as detergent, paper and food [14]. *A. oryzae* IFO-30103 was proved to be a very good source of hyperactive α-amylase [15]. Therefore, there is a dire need for strain development to engineer the organisms according to our needs.

In nature there is a vast variety of naturally occurring microbes capable of producing enzymes which are exploited for industrial needs. Strain improvement is the basic part of process development, generally aiming at decrease of production costs. Strain development techniques aim at increasing enzyme and biomass yields along with enhanced physiological properties efficient consumption of a variety of industrially relevant substrates. The techniques used for strain improvement are directed evolution, recombinant DNA technology, dominant selection and random mutagenesis [16]. Different approaches used for fungal strain development generally comprised of one or more of the following: (i) the use of chemical and physical mutagens to induce random mutations in the genome, (ii) application of parasexual and sexual reproduction to obtain novel recombinants and (iii) genetic engineering to introduce novel material in the fungal genome or to inactivate unwanted genes. These methods can be applied either separately or in different combinations [17]. While acquiring the secreted industrial enzymes, the popular technique is genetic engineering whereas, for the production of edible products and biocontrol agents for field release the non-recombinant approach involving traditional mutagenesis is preferably chosen. Recombinant technology has been effectively used in strain improvement over the years but, since the processes used by the food industry are under strict regulations and monitored closely. The use of recombinant DNA technology is not favored in many developed countries. It is for this reason that classical strain improvement is of immense importance for academic research [18].

Random mutagenesis is extensively used in the food industry for the classical strain improvement purposes [19]. The basic mechanism underlying this approach is the introduction of random mutations into the genome of interest, categorization of a huge number of variants, and screening of strains with the desired quality for further use. This approach surpasses others as very little knowledge is required and it is very useful for exclusion of specific characteristics where direct selection is not possible. However, the use of dangerous chemicals, mutation bias and hot spots are the drawbacks of this approach. It requires extensive screening and characterization of a large number of survivors [18]. Strain improvement is trial and error process comprising of a procedure [20].

Gamma rays are used as a very effective mutagenic agent for strain improvement. Different sources are used for the purpose of irradiation. The γ-rays of Co-60, UV and NTG have been used for the mutation of fungal strains to induce the hyper-production of cellulases [21]. Irradiation by gamma rays may cause some mutations to the genes of cells through the DNA repair mechanisms within cells [22].

The novelty of current study is that we for the first time have presented a five steps sequential screening protocol to develop potent Koji mutant for the hyper production of thermostable enzyme. Hence, main focus of the envisaged project was to develop a process to improve productivity and thermal stability of Koji’s α-amylases simultaneously through random mutagenesis by using high ionizing γ-rays for the persistent mutations.

## Results

A process has been developed for the screening of fungal mutants’, hyper producer of thermostable α-amylases, by random mutagenesis. The super Koji (*A. oryzae* cmc1), which is a transgenic strain, was subjected to gamma rays treatment to generate mutants. In this project we screened a total of 52 mutant strains for the desirable attributes i.e. hyper production of alpha amylases with enhanced thermal stability. After extensive screening and careful analysis we were able to select a mutant Koji strain which was best suited for maltose syrup production (saccharification) from corn starch liquor. Moreover, the mutant was also tested for toxins production, if any.

### Random mutagenesis of super Koji

After treatment by gamma rays the untreated and treated *A. oryzae* samples were spread on the culture plates containing 0.4% (*w/v*) Triton X-100 and incubated at 30 °C for 72 h. The viable colonies i.e Colony Forming Unit (CFU ml^-1^) were counted and found that all gamma rays doses i.e. 60, 80, 100, 120 & 140 KRad (0.60, 0.80, 1.00, 1.20 & 1.40 KGy) exhibited a log kill of 3 or more (Table 1).

**Table 1:** Effect of gamma rays on the survival of super Koji (*A. oryzae*) grown on Potato Dextrose Agar (PDA) plates in the presence of 0.4% Triton X-100

Dose (kGy)	CFU ml^-1^	Log CFU ml^-1^	% Survival	% Kill	Log Kill
Parent Koji	3,450,000	6.538	100	−	−
0.6	3,167	3.501	0.092	99.908	3.037
0.8	2,666	3.426	0.077	99.923	3.112
1.0	1,167	3.067	0.034	99.966	3.471
1.2	500	2.699	0.014	99.986	3.839
1.4	167	2.223	0.004	99.996	4.315

Where: CFU ml^-1^ is an average of triplicate values

CFU ml^-1^ = Viable count ml^-1^ of sample spread on the plate × Dilution factor

% Survival = CFU ml^-1^ of irradiated÷ CFU ml^-1^ of control ×100

% Kill = 100 - % Survival

Log kill = log CFU ml^-1^ of control - log CFU ml^-1^ of irradiated fungus

Gamma source= Cesium 137

### Process development for mutants screening

#### 1^st^ Step: *Resistance to 1% Triton X-100*

Well grown colonies resistant to 1% (*w/v*) Triton X-100 were carefully picked and purified. We selected 12 colonies each from 0.60, 0.80, 1.00 KGy dose and 11 and 5 from 1.20 and 1.40 KGy dose, respectively.

#### 2^nd^ Step: *Hyper production of α-amylase on Solid Fungal Growth Medium (SFGM)*

As a result of inoculation of mutants on the SFGM the extracellular α-amylase enzyme was produced by each strain, which digested the starch present in the culture plate and the clearing zone index (CI) was calculated. On the basis of CI 23 mutants were screened out of the initial 52. The CI varied in a range on 2.1-5.8 with mutant M- 120(7) and M-100(12) having the smallest and largest clearing zone index, respectively (Additional file 1: Table S1).

#### 3^rd^ Step: *Resistance to 2-deoxy-D-Glucose*

**Fig. 1:**
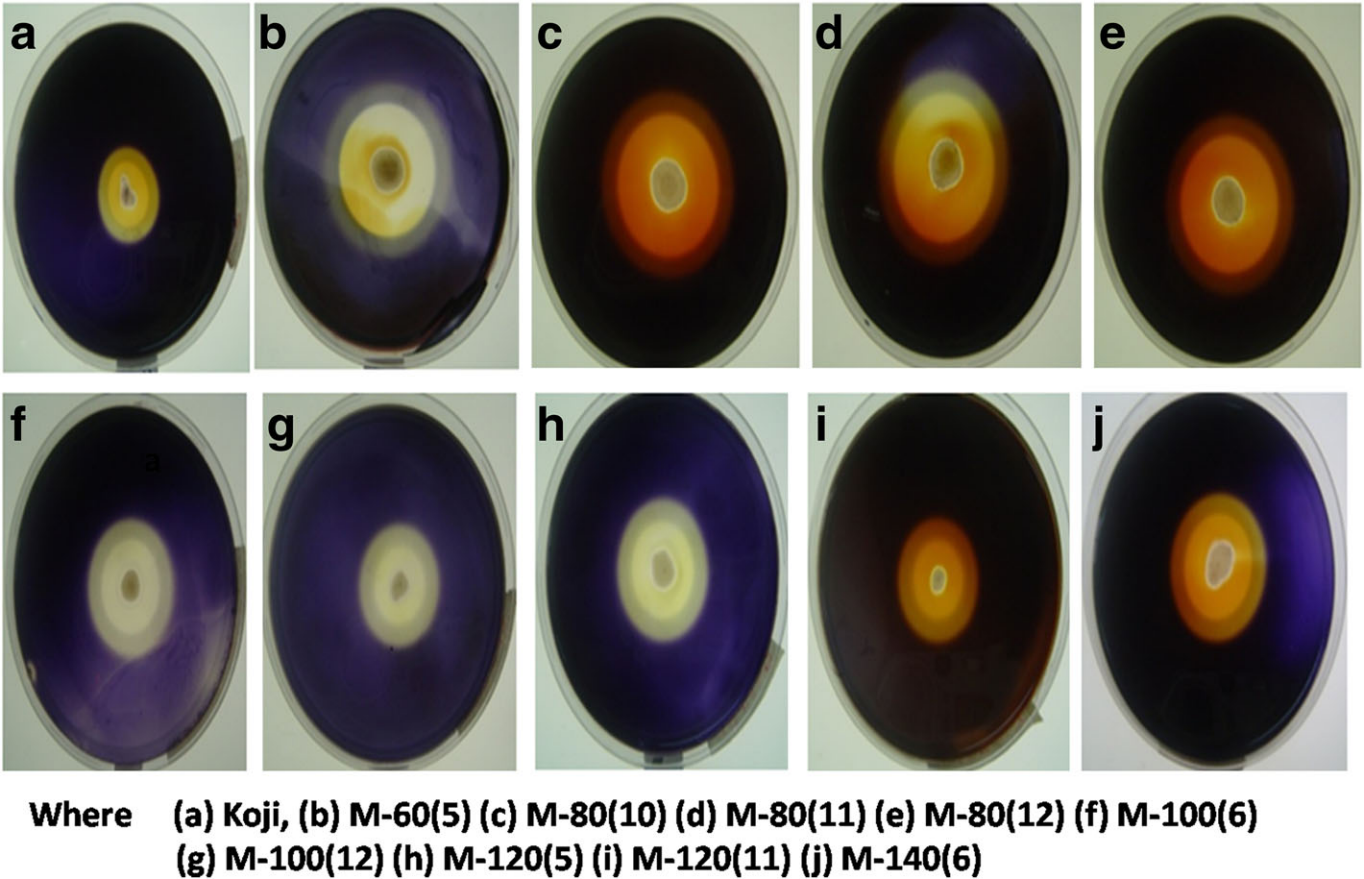
Production of α-amylases by A. oryzae cmc1 mutants on agar plates containing 2-Deoxy D-glucose (0.1%, w/v) & 0.2% Triton X100. **a** Koji, **b** M-60(5), **c** M-80(10), **d** M-80(11), **e** M-80(12), **f** M-100(6), **g** M-100(12), **h** M-120(5), **i** M-120(11) and **j** M-140(6)

Twenty three (23) mutants screened at previous stage were then subjected to catabolite repression i.e. resistance to 2-Deoxy-2-D-Glucose. The potent mutants were selected on the basis of CI and nine (9) mutants were finalized for next level of screening (Fig. 1). The mutant M-120(5) had highest CI of 3.40, whereas the parental strain showed CI of 2.0 (Table 2).

**Table 2:** Clearing Zone Index for the Production of α-Amylases by *A. oryzae* cmc1 Mutants on Agar Plates containing 2-Deoxy D-Glucose (0.1%, *w/v*) & 0.2% Triton X100

Sr. No	Mutant no.	Halo zone + Colony diameter (cm)	Colony diameter (cm)	Clearing zone index
1	Parent Koji	1.2±0.07	0.60±0.04	2.00
2	M-60(2)	1.3±0.08	0.50±0.03	2.60
3	M-60(4)	1.1±0.06	0.60±0.04	1.83
4	M-60(5)	1.8±0.14	0.65±0.04	2.77
5	M-60(7)	0.8±0.06	0.70±0.04	1.14
6	M-60(10)	1.1±0.08	0.60±0.03	1.83
7	M-60(11)	0.9±0.06	0.60±0.04	1.50
8	M-80(6)	1.3±0.05	0.60±0.03	2.16
9	M-80(9)	1.2±0.05	0.50±0.03	2.40
10	M-80(10)	1.7±0.08	0.55±0.03	3.09
11	M-80(11)	1.5±0.06	0.60±0.04	2.50
12	M-80(12)	1.6±0.07	0.60±0.03	2.66
13	M-100(2)	1.4±0.06	0.70±0.04	2.00
14	M-100(3)	1.5±0.08	0.65±0.04	2.30
15	M-100(4)	0.9±0.04	0.40±0.02	2.25
16	M-100(6)	1.9±0.09	0.60±0.04	3.16
17	M-100(7)	1.7±0.08	0.70±0.04	2.42
18	M-100(9)	1.1±0.05	0.50±0.03	2.20
19	M-100(12)	1.9±0.09	0.65±0.04	2.92
20	M-120(4)	1.3±0.08	0.60±0.03	2.16
21	M-120(5)	1.7±0.10	0.50±0.03	3.40
22	M-120(10)	1.3±0.07	0.55±0.03	2.36
23	M-120(11)	1.6±0.09	0.60±0.03	2.67
24	M-140(6)	1.6±0.09	0.60±0.04	2.67

Clearance zone index (CI) = (halo zone diameter + colony diameter) /colony diameter. Data presented are average values ± SD of *n* = 3 experiments

#### 4^th^ Step: *Hyper production of α-amylase on Liquid Fungal Growth Medium (LFGM)*

Wet cells density of the innoculi of parental and its mutant derivatives was calculated to ensure the equal amount of cells transfer for the production of α-amylase (Additional file 1: Table S2). The inoculum concentration of wet cells used was 0.3% (*w/v*).

Total α-amylases activity, extracellular proteins and specific activity of all the nine mutants was calculated. Three mutant Koji strains i.e. M-80(10), M-100(6) & M-120(5) gave about 4 fold hyper production of α-amylases, whereas, about 3-fold increase was for M-100(12), M-120(11) & M-140(6) mutants. The Koji strain M-100(6) produced highest amount of α-amylase (26.77 U ml^-1^), which was about 4 fold higher than control (6.55 U ml^-1^). Specific activity of α-amylases produced by all mutants was higher than the parental strain (31.75 Umg^-1^) and highest value (370.73 Umg^-1^) was observed for M-100(6) mutant and was 11.7 fold higher (Table 3).

**Table 3:** Production of α-Amylases by 2-Deoxy D-Glucose Resistant Mutant Derivatives of *A. oryzae* Grown under Submerged Conditions on Soluble Starch

Sr. #	γ-ray Exposure	α-amylase (U ml^-1^ )	Protein (mg ml^-1^ )	Specific activity (U mg^-1^ )
1	Parental Koji	6.55±0.45	0.21±0.011	31.8
2	M-60(M5)	15.49±1.05	0.14±0.007	114.5
3	M-80(10)	26.65±1.81	0.11±0.006	251.5
4	M-80(11)	9.76±0.57	0.14±0.007	70.0
5	M-80(12)	4.92±0.29	0.1±0.005	48.9
6	M-100(6)	26.77±1.39	0.07±0.004	370.7
7	M-100(12)	17.83±0.93	0.05±0.003	332.7
8	M-120(5)	26.22±1.36	0.12±0.006	215.2
9	M-120(11)	21.05±1.10	0.18±0.009	118.0
10	M-140(6)	19.47±1.01	0.26±0.013	75.7

Data presented are average values ± SD of *n* = 3 experiments

#### 5^th^ Step: *Hyper thermal stability of α-amylases*

**Fig. 2:**
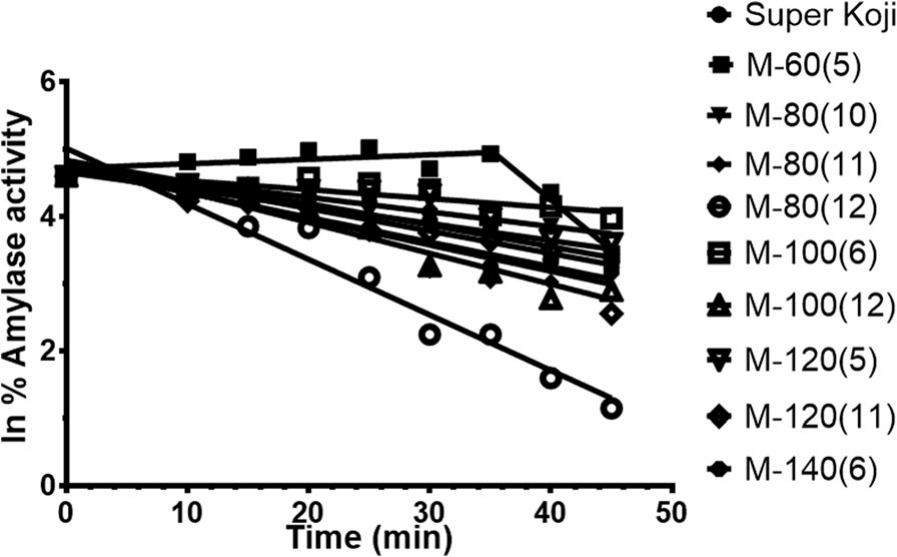
Pseudo 1st order plots for irreversible thermal inactivation of α-amylases from mutant strains of A. oryzae cmc1 at 55 °C

Pseudo first order plots were applied to determine half-life (t½) at 55 °C of α-amylases produced by the Koji mutant strains (Fig. 2). Irreversible thermal stability of M-100(6) α-amylase was highest i.e. 2.5 fold among the mutants. The α-amylases of M-80(10) and M-120(5) were about 1.5 fold more stable than parental strain. Whereas, enzyme stability of M-80(12) was about 2.4 fold decreased. Surprisingly M-60(5) amylase showed activation trend up to 30 min of incubation (Table 4).

**Table 4:** Irreversible Thermostability of α-Amylases from 2-Deoxy D-Glucose Resistant Mutant Derivatives of *A. oryzae* at 55 °C

Sr. #	γ-ray Exposure	*K*_d_ (min^-1^ )	t½ “Half Life” (min)
1	Parental Koji	-0.033419	20.74
2	M-60(5)	+0.006681 -0.148948	103.79^td^ 4.65
3	M-80(10)	-0.025435	27.25
4	M-80(11)	-0.039135	17.71
5	M-80(12)	-0.082685	8.38
6	M-100(6)	-0.013307	52.09
7	M-100(12)	-0.045898	15.10
8	M-120(5)	-0.021796	31.80
9	M-120(11)	-0.036753	18.86
10	M-140(6)	-0.032123	21.58

*K*_d_ (first order rate constant of inactivation), t½ (half-life) = 0.693/*K*_d_. Activation trend was observed for M-60(5),t_d_ (doubling time) = 0.693/*K*

### Toxin analysis

**Fig. 3:**
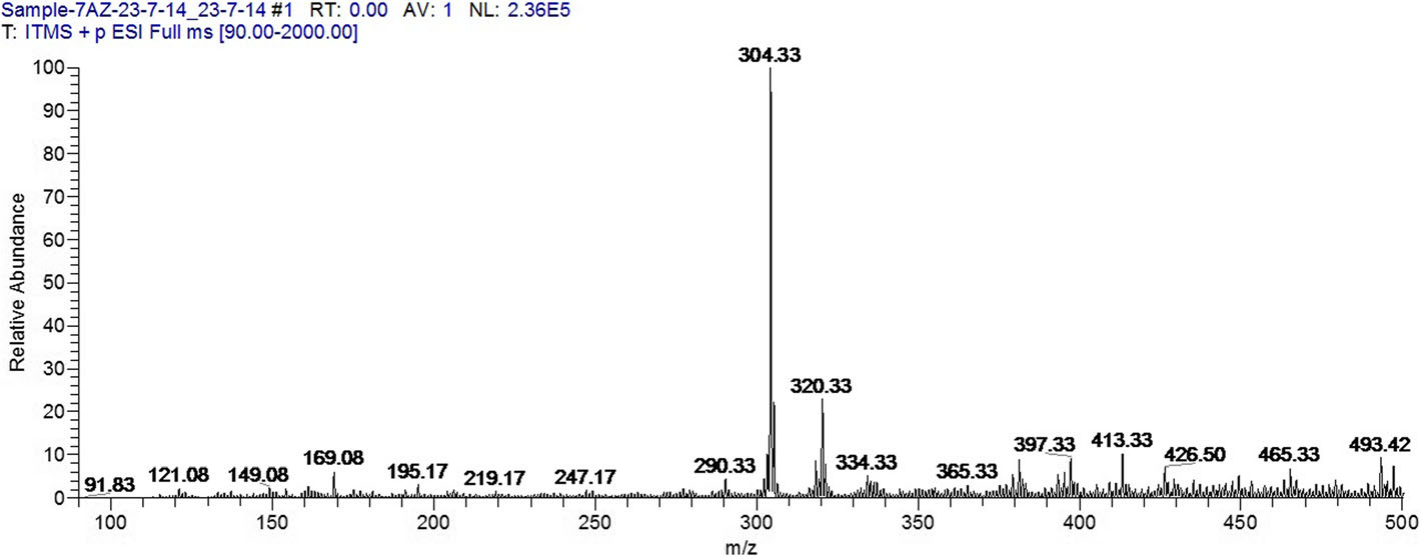
Full scan mass spectrum of M-100 (6) using direct syringe pump

**Fig. 4:**
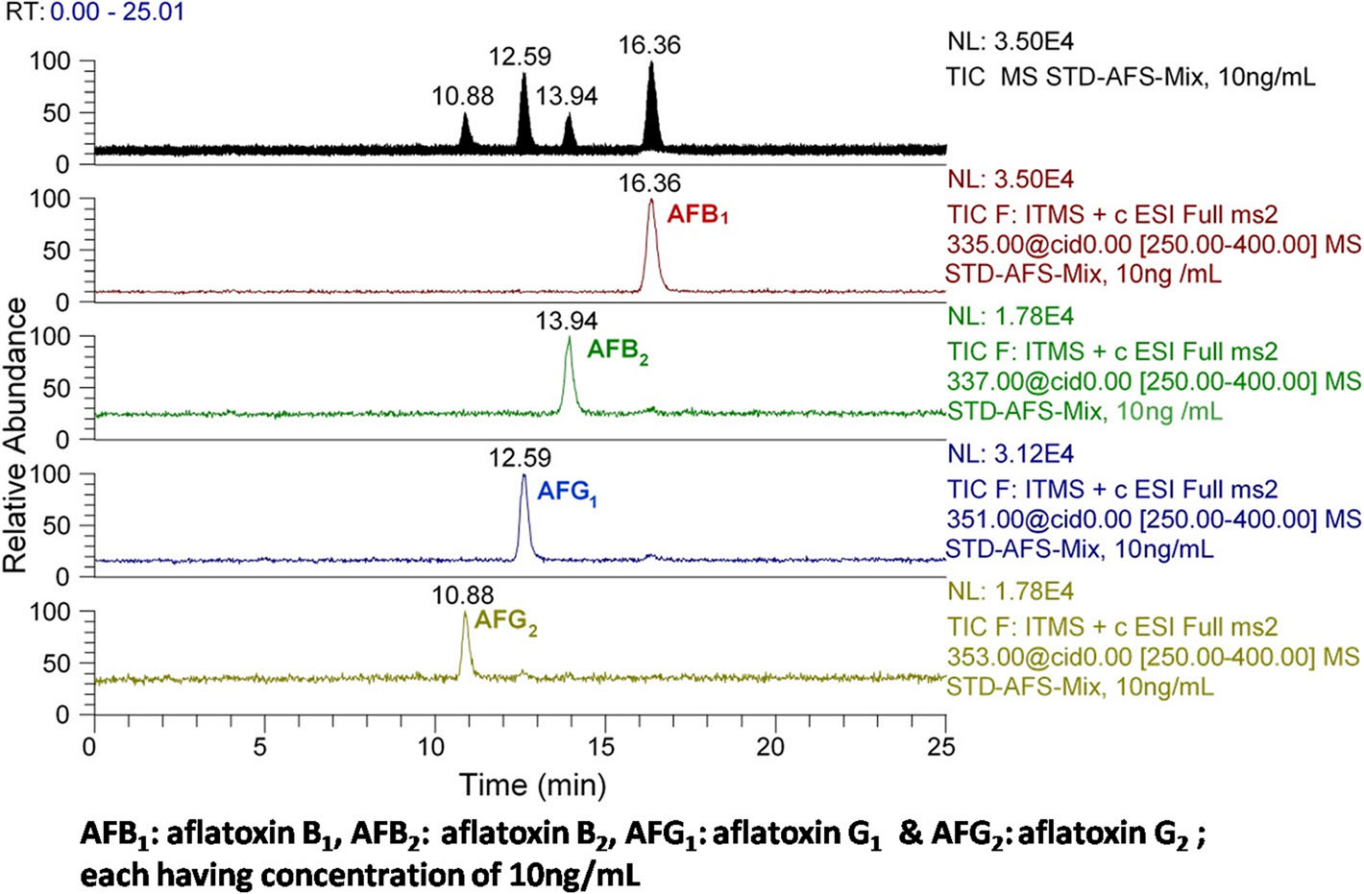
Mixture of aflatoxins standards chromatographed on LCMS/MS

**Fig. 5:**
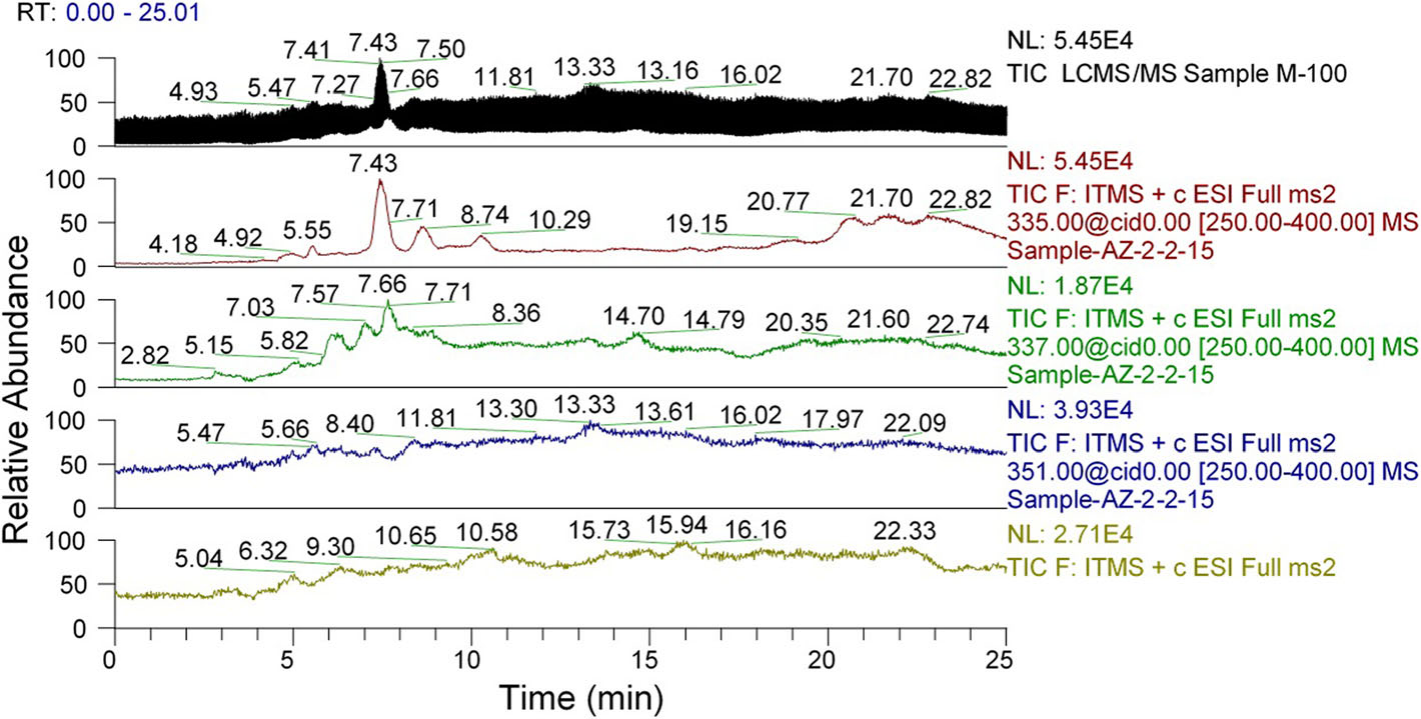
LCMS/MS scans of the extract derived from M-100(6)

The crude extract was analyzed for aflatoxin production. The full scan and selective ion monitoring showed no production of aflatoxins by the mutant (Fig. 3). The results were further verified by conducting analysis on LC-MS/MS using previously developed and published method. The sample scans were compared with standards (Figs. 4 & 5), which confirmed that aflatoxins were not present in α-amylase solution of the parental as well as the mutant strain.

### FESEM of Koji (*A. oryzae*)

**Fig. 6:**
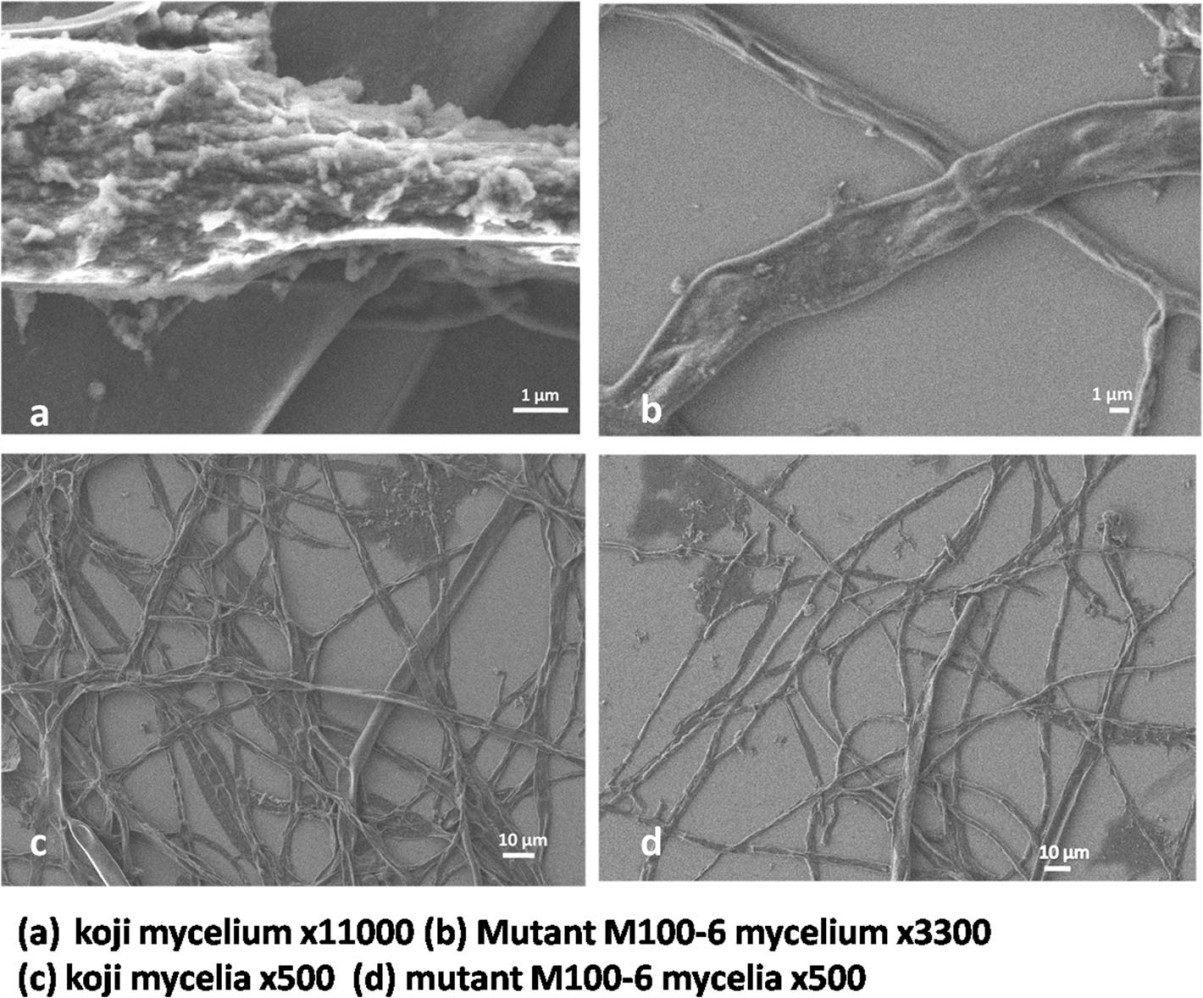
Scanning Electron Micrograph of the control strain koji and M-100(6) mycelia. **a** koji mycelium x11000, **b** Mutant M-100(6) x3300, **c** koji mycelia x500 and **d** Mutant M-100(6) mycelia x500

To assess effect of gamma rays on morphology of the fungal mycelium FESEM was done. The mycelium becomes more compact/ rigid after application of gamma rays. The images of control Koji strain were successfully taken up to resolution of x11,000, while for M100-6 mutant strain the maximum resolution was x3,300 (Fig. 6).

### Production of alpha amylase in bioreactors

**Fig. 7:**
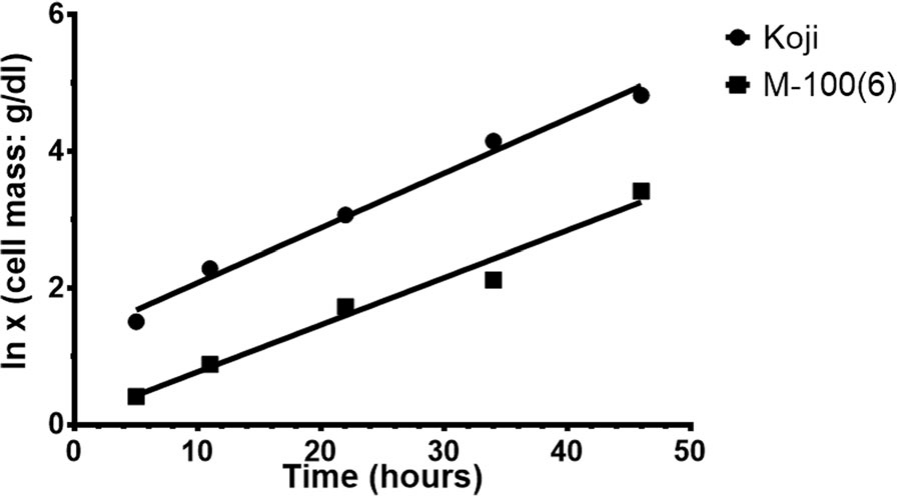
First order plot for specific growth rate of cell mass formation

The productivity of α-amylase by M-100(6) strain in 10L fermenter was maximum (4004 U dl^-1^) after 23 h. The mutant strain was more resistant as compared to parental strain. Specific rate (*q*_p_) of α-amylase production of mutant strain (33.29 U g^-1^ h^-1^) confirmed that the mutant was more efficient in α-amylase production than the parental strain (11.89 U g^-1^h^-1^). Specific activity of α-amylases produced in 10L fermenter by mutant and parental strain was (2461 Umg^-1^) and (2452 Umg^-1^), respectively (Table 5 and Fig. 7).

**Table 5:** Growth Kinetics of α-Amylase Production by Mutant Derivative of Super Koji (*A. oryzae* cmc1) in 10L Fermenter Grown under Submerged Condition on Soluble Starch (2% *w/v*) at 30 °C

Strain	α-Amylase (U dl^-1^)	Protein (mg dl^-1^)	Cell mass (g dl^-1^)	*μ* (h^-1^)	t_d_ (h)	*Y*_p/x_ (U g^-1^)	*q*_p_ (U g^-1^ h^-1^)
Parent Koji	3181	1.297	21.4	0.08	8.66	149	11.89
Mutant-100(6)	4004	1.627	8.3	0.069	10.05	482	33.29

Where: *μ* (Specific growth rate); t_d_ (Biomass doubling time) =ln2/*μ*, *Y*_*p*/x_ = Product yield coefficient with respect to cell mass; *q*_p_ (Specific rate of product formation) =*Y*_*p*/x_× *μ*

## Discussion

*A. oryzae* isolates are generally used in variety of industrial processes [12]. Strain development plays important role in making a process more feasible. Random mutagenesis is a well-known approach for the improvement of microbes [16]. A process was developed to improve transgenic fungal strain *A. oryzae* cmc1 (Super Koji). After extensive screening procedure, we have successfully developed a mutant Koji strain M-100(6), which is hyper producer of thermostable α-amylases.

Initially we screened the mutants stock based on resistance to Triton x-100 (1% *w/v*), which is a detergent, with the idea that mutants resistant to this extreme environment may be having thermostable enzymes. Detergents are found to affect the fungal metabolism and also exhibit inhibitory effect on the overall growth of fungi. Triton-x100 restricts the colony size; therefore, it hinders the merging of two colonies on a culture plate [23–25]. This was found very effective in our initial screening for the mutants as it restricted the growth and as a result we were able to get separate individual colonies for further propagation of culture.

In solid state culture the enzyme production is determined by measuring the clearing zone around the fungal colony and a larger halo zone is indicative of higher enzyme production. Halo zone diameter is a reliable parameter for strains screening [17, 26, 27]. Clearing zone index (CI) is an accurate way to evaluate enzymes productivity by the mutants. CI gives a better insight of the enzyme production in accordance with the size of the colony, hence growth of the organism [21]. Catabolite repression is the phenomenon in which the enzyme production is hindered/ lowered in the presence of the catalysis product. This is caused due to the existence of microscopic gradients between cell aggregates or alteration in cell permeability to sugars [28]. For the screening of repression resistant mutants glucose analog 2–deoxy-D-Glucose is used [22, 29]. A decrease in the size of clearing zone is reported due to the presence of sugars in media [30]. Catabolite repression approach was used to get the mutants and we isolated the mutants which showed bigger CI despite the presence of 2DG indication hyper production of the desired enzyme. Improved thermal stability of the β-glucosidase (BGL) enzymes by random mutagenesis and in the presence of a glycosylation inhibitor (2-Deoxy-D-glucose) had been reported for *Termitomyces clypeatus* [31]. The stability of BGL from mutant derivative of *Cellulomonas biazotea* NIAB 442 against 4 M urea was improved [32].

Specific activity is an index to determine the purity of the enzyme in a mixture. A high specific activity is indicative of the higher concentration of the desired enzyme amongst the total proteins of the system [33]. Apart from the high per unit activity the selected mutant had a specific activity of 370.73 Umg^-1^, which was ~12 times higher as compared to the parental strain. This showed that the mutant was capable of producing the desired enzyme a lot more than control strain. The specific activity of partially purified amylase of *A. awamori* was 69.7 Umg^-1^ [33].

Thermal stability of enzymes is the most important parameter to increase the economy of industrial process [26, 34]. Temperature is the most important factors which markedly influences the enzyme activity. The thermal stability of mutant M-100(6) alpha-amylase at 55 °C was increased along with the per unit activity. At higher temperatures the stability of the enzyme decreases. The α-amylases from *Bacillus licheniformis* AT70*, A. Niger* strains*, R. oryzae* FSIS4*, Fusarium* sp. and *Laceyella sacchari* TSI-2 have been reported to retain residual activity of 70-90% at temperatures between 40-60 °C [34–38]. The α-amylase produced from thermophilic fungal strains STG3E and STG6E showed maximum amylase activity at 55 °C after 10 min incubation [26]. The thermostability of mutant amylase was enhanced and the half-life was increased to 2.5 folds.

Javed et al., 2018 reported that Gamma rays induced physical mutations in *A. niger* improved the properties of BGL. Physiochemical and thermodynamic characterization of extracellular BGL displayed that mutagenesis did not affect the physiochemical properties of the BGL enzyme to an acceptable level, such as temperature optima, pH optima and molecular mass, whereas, it significantly improved the catalytic efficiency for cellobiose hydrolysis. Furthermore, the thermostability of the mutant BGL was more than the parent enzyme. Moreover, we found that γ-rays induced 8 point mutations within the *bgl* gene of mutant strain as compared to parental strain. Out of these 8 mutations 2 were true substitutions (T54M and T225M), 2 were semi-conserved (N259S and S264N) and 3 were conserved substitutions (M460V, N513D and D638N), while one was silent mutation (N211N). Both semi conserved mutations (N259S and S264N) were found very close to the enzyme active site. *A. niger* mutated BGL gene analysis confirmed that out of 8 transitions were within the exons, A/T to G/C transitions were predominant (37.5%), while no transition within introns/any transversion/ tandem double base substitution or base insertions/deletions [39]. These transitions might be due to tautomerization reactions [40] induced by gamma radiations. Similar reports are found for the mutation spectrum induced by gamma rays in *Pleurotus ostrreatus* manganese peroxidase gene [41] and in lambda phage and prophage DNA [42]. Hence, we consider the activation and thermostabilization of super Koji mutant 100(6) α-amylase was might be due to transitions in the amylase gene.

Aflatoxins are a group of difuranocoumarin derivatives that consist of a coumarin and a double-furan-ring of molecule usually. These are very toxic, teratogenic, mutagenic and carcinogenic compounds [43]. *A. oryzae* is closely related to *A. flavus* phylogenetically, thereby, sharing very high similarity in their genome size and amino acid sequences. This relationship of *A. oryzae* with *A. flavus* has led to extensive screening of the toxic potential of *A. oryzae*, so far no *A. oryzae* isolate is reported to produce aflatoxin. One of the attributes of *A. oryzae* that is responsible for deciding its fate for industrial use in comparison to *A. flavus* is that *A. oryzae* does not produce aflatoxins despite having a very similar gene cluster.

LC-MS/MS, an extremely specific and highly sensitive technique for testing food products with superior accuracy was used to detect aflatoxins in our samples. LC-MS/MS is a powerful tool for analyzing mycotoxins specifically, as it aides the laboratories to do the qualitative and quantitative analysis of multiple mycotoxins simultaneously, in less time, with greater accuracy [43, 44]. The results confirmed there were no aflatoxins in the amylases produced by the mutant M-100(6). Hence, our mutated Koji strain and its enzymes are safe for application in food and feed industry.

The FESEM scans depicted the mutant M-100(6) to be more stable and stiff as compared to the parental strain. It was found to withstand harsh conditions and therefore, the mycelia remained intact after treatment for sample preparation for FESEM. The growth kinetic parameters of the mutant strain determined using 10L fermenter confirmed that the strain was hyper producer of thermostable amylases and have high potential for industrial application.

## Conclusion

Here we conclude microbial strains especially fungi can be improved simultaneously to enhance thermostability and productivity of extracellular enzymes through high ionizing physical mutagens (γ-rays) by using our five step screening protocol. The potent super Koji mutant M-100(6) was resistant to Triton x-100 and catabolite repression. Mutagenesis made the Koji strain more specific in α-amylase production (~ 12 fold increases) and enhanced the enzyme irreversible thermal stability by 2.5 folds at 55 °C. The increase in production and thermostability of α-amylases indicated that γ-rays might have mutated the α-amylases structural, as well as, the regularity genes. The mutagenesis made Koji’s mycelium more rigid and did not trigger the aflatoxins production. Due to variability in microbial strains resistance to survive at high level of detergents and temperature, it is pertinent to mention that the concentration of Triton x-100 and temperature will be required to be adjusted for each organism. We found the developed thermostable α-amylases of super Koji mutantM-100(6) has immense potential and is safe for application in food and feed industry.

## Methodology

### Microbial strain

The super Koji *A. oryzae*, a derivative of wild-type strain RIB40, which was developed through expression cloning of *cmc*1 gene of *A. aculeatus* [45] was obtained from Industrial Enzymes & Biofuels Group, Industrial Biotechnology Division, National Institute for Biotechnology and Genetic Engineering (NIBGE), Faisalabad, Pakistan.

### Fungal growth medium composition

The Koji strain was maintained on Solid Fungal Growth Medium (SFGM). The ingredients of SFGM were dissolved/mixed gradually in about 750 ml distilled water as mentioned below: 3g NaNO_3_, 50 ml Salt solution (% *w/v*: 2.6 KCl, 2.6 MgSO_4_.7H_2_O, 7.6 KH_2_PO_4_); 01ml Trace element solution (% *w/v*: 0.11 Mo_7_O_24_.4H_2_O, 1.11 H_3_BO_3_, 0.16 CoCl_2_. 6H_2_O, 5 EDTA, 0.5 FeSO_4_.7H_2_O, 0.5 MnCl_2_.4H_2_O and 2.2 ZnSO_4_.7H_2_O), 10 g glucose and 15 g agar. The pH of the medium was adjusted to pH 6.5 by 1M NaOH/HCl. Afterward the volume of SFGM was made up one litre with distilled water.

In Liquid Fungal Growth Medium (LFGM), for the enzyme production, the concentration of salt and trace elements was the same as in SFGM; whereas, 1% (*w/v*) Polypeptone and 10 mM ammonium tartarate were used as nitrogen source instead of NaNO_3_. The *A. oryzae* was grown on different carbon sources (2% *w/v*) at 30 °C with shaking speed 150 rpm [45].

### Culture maintenance & preparation of petri-plates

The Koji strain was maintained on SFGM in petri plates and was stored at 4 °C as described [45]. Briefly, approximately 25-30 ml autoclaved media was poured aseptically into autoclaved Pyrex glass petri plates, which were then allowed to solidify. Later on, plates were inoculated aseptically by loop full of super Koji *A. oryzae* spores. The plates were incubated at 30 °C for about 12 to 15 days till the proper growth i.e. yellow spore formation. Afterward, the plates were sealed properly by paraffin and stored at 4 °C. The culture was refreshed once a month.

### Inoculum preparation

The innoculum development was carried out as described by Rashid et al. [45]. One hundred ml of LFGM was added per 250 ml Erlenmeyer flasks. About 8-10, properly washed glass beads (*ø 8.0 mm*) were added to each flask to break the fungal mycelia. Appropriate amount of autoclaved glucose stock (30% *w/v*) was added aseptically in the growth medium to achieve final glucose concentration 2% (*w/v*) as a carbon source. The super Koji spores (2-3 platinum wire loop-full) were aseptically transferred to the flasks, which were then incubated in orbital shaker at 30 °C, 150 rpm. The fungus was grown for about 36 h.

To estimate the wet cells weight or packed cell mass of the inoculums, samples in triplicate were removed aseptically by using autoclaved one ml micropipette tips (Tips were cut to broaden their neck). Then, samples were centrifuged for 5 min and supernatant was discarded. The weight of wet cell mass was determined by subtracting the weight of empty eppendorf tube from the cell mass containing tube.

### α-Amylase plate assay & clearing zone index

The screening of α-amylase hyper producing mutants was done by plate assay. The control super Koji strain of *A. oryzae* and its mutant derivatives were transferred onto SFGM plates containing soluble starch (1% *w/v*) and Triton-X100 (0.25% *v/v*) as colony restrictor. The cultures were grown for exact 6 days and analyzed for the clearing zone formation. Moreover, the α-amylase activity was confirmed by using iodine staining method [22]. The plates were treated with iodine solution (*w/v*: Iodine 0.33% & KI 0.66%; dissolved KI in 5 ml water, then mixed iodine and finally volume was made up to 100 ml) for about 40 min and then washed (2–3 times) with distilled water. The undigested starch gave the blue color. The diameter of clearing/enzyme activity zone and fungal colony was measured at two dimensions. The clearing zone index (CI) for α-amylase activity was estimated based on the principle described by Paul and Sinha [46] for the phosphorus solubilisation index. The CI was determined by using the formula:

$$ \mathrm{Clearing}\ \mathrm{Zone}\ \mathrm{Index}\ \left(\mathrm{CI}\right)=\frac{\mathrm{Halo}\ \mathrm{zone}\ \mathrm{diameter}+\mathrm{Colony}\ \mathrm{diameter}}{\mathrm{Colony}\ \mathrm{diameter}} $$

### α-amylase assay

The α-amylase (100 μl) was incubated for 30 min at 45 °C in the presence of one ml soluble starch (1% *w/v*) and one ml 50 mM sodium acetate buffer pH 5; the reaction was quenched by placing the tubes in boiling water for 5 min. The product released was determined by measuring the reducing sugars through DNS-method [22]. Briefly, two ml of DNS reagent was mixed with appropriate amount (0.5-2.1 ml) of quenched reaction mixture (QRM) and volume was made up to 4.1 ml. Then boiled for 10 min and after cooling of the reaction mixture, change in absorbance (ΔA°) was measured at 550 nm. Glucose standard factor was determined from the glucose standard curve and was 2.02 μmole.

One unit α-amylase activity was defined as the amount of enzyme required to release one μmol of reducing groups (calculated as glucose equivalents) min^-1^ from soluble starch at 45 °C, pH 5. The α-amylase activity units were calculated by using the formula:

$$ \mathrm{Units}/\mathrm{ml}/\min =\frac{{\Delta \mathrm{A}}^{{}^{\circ}}\times \mathrm{G}.\mathrm{S}.\mathrm{factor}\ (2.02)\times \mathrm{Dil}.\mathrm{factor}\times \mathrm{Total}\ \mathrm{reaction}\ \mathrm{mixture}\ \left(2.1\ \mathrm{ml}\right)}{\mathrm{Enzyme}\ \left(0.1\ \mathrm{ml}\right)\times \mathrm{Time}\ \left(30\ \min \right)\times \mathrm{QRM}\ \mathrm{for}\ \mathrm{DNS}\ \mathrm{assay}\ \left(0.5\ \mathrm{ml}\right)} $$

Where,

ΔA° = Change in optical density (OD) at 550 nm = Experimental ‘OD’ - Blank ‘OD’.

Glucose Standard Factor = G.S. factor = 1.0 OD = 2.02 μmole glucose

QRM = Quenched reaction mixture

### Protein estimation

The extracellular proteins were determined by Bradford assay and BSA was used as a standard [45].

### Random mutagenesis & determination of 3.0 log kill of *A. oryzae*

The freshly prepared innoculum of super Koji (*A. oryzae*) was aseptically distributed in six sterile fifty ml falcon centrifuge tubes (fifteen ml tube^-1^) and irradiated with gamma rays by using Caesium-137 (Cs-137) source. The fungus was treated with various γ-rays doses: 0.6, 0.8, 1.0, 1.2 & 1.4 kGray in a gamma cell radiation chamber (Mark-IV Irradiator/Gamma Cell-220) available at Nuclear Institute of Agriculture and Biology (NIAB), Faisalabad, Pakistan [22, 47, 48].

The control and irradiated Koji samples were serially diluted (50 - 600 fold). Viable cell counts was made through the determination of colony forming units (CFU ml^-1^), which was achieved by spreading the serial dilutions of control (un-treated) and irradiated cells from each dose on SFGM culture plates in the presence of 0.4% (*v/v*) Triton X-100 as a colony restrictor. The plates were incubated at 30 °C for about 6 days so that colonies may grow appropriately. The colony forming units CFU ml^-1^ was calculated as follows:

$$ \mathrm{CFU}/\mathrm{ml}=\frac{\mathrm{Average}\ \mathrm{viable}\ \mathrm{counts}\ }{\mathrm{Sample}\ \mathrm{volume}\ \left(\mathrm{ml}\right)}\times \mathrm{Dilution}\ \mathrm{factor} $$

To select the potent gamma ray mediated mutants i.e. having persistent mutations, 3.0 log kill of Koji cells (99.99% kill) was determined. The γ-rays doses having at least 3 log kill were used for the mutants selection.

### Process development for potent Koji mutants screening

#### 1^st^ Screening: Resistance to 1% (*w/v*) triton X-100

Initially main stock of potent Koji mutants (Total=54) was made on the basis of resistance to 1% (*w/v*) Triton X-100. Briefly, the irradiated super Koji variants were plated on SFGM plates containing Triton X-100 and grown under the same conditions as mentioned before. Well defined mutated Koji colonies (about 12 per doze) resistant to 1% Triton X-100 were selected, purified and propagated for further use.

#### 2^nd^ Screening: Hyper production of α-amylase on SFGM

The preliminary selected mutants, resistant to 1% Triton X-100, were grown on SFGM plates containing 1.0% (*w/v*) soluble starch as a substrate. Moreover, 0.3% (*v/v*) Triton X-100 was added to restrict the growth of colonies, so that cell focuses only on the production of α-amylases. The plates were incubated at 30 °C for 115 h and examined for clearing zone formation. Afterwards, plates were stained for zymographic analysis of α-amylase activity. The diameters of halo/α-amylase activity zone and fungal colony were measured to estimate the CI as described earlier.

#### 3^rd^ Screening: Resistance to 2-deoxy-D-glucose (Catabolite Repression)

Next, screening of α-amylase hyper producer mutants was done based on 2-deoxy-D-Glucose (2DOG) resistance i.e. a catabolite repressor. The mutants after 2^nd^ screening were grown on agar SFGM plates containing (*w/v*): 1% starch, 0.1% 2-deoxy-D-Glucose and 0.2% Triton X-100 [49]. The CI for α-amylase was estimated and 2DOG resistant Koji mutants having highest CI were selected.

#### 4^th^ Screening: Hyper production of α-amylase on LFGM

The 2DOG resistant Koji mutants were further screened on the basis of α-amylase hyper production under submerged growth conditions. The mutants after 3^rd^ screening were grown on 2% (*w/v*) soluble starch in 250 ml Erlenmeyer flasks and one hundred ml of LFGM containing 6-8 glass beads was used per flask.

Inoculums of all the selected mutants were prepared and cell mass densities were estimated as mentioned before. Then, equal amount of mutant’s inoculum (0.3% *w/v*) was transferred in the flasks containing the LFGM for the production of α-amylases. The batch culture was grown for 72 h; then the crude enzyme extracts were filtered through muslin cloth and centrifuged at 10,000 rpm (15,300 **×***g*) at 4 °C for 15 min.

The supernatants were separated and processed for the estimation of total proteins and α-amylase activity in crude extracts. Afterwards, the super Koji mutants having highest production and specific activity of α-amylase were selected.

#### 5^th^ Screening: Hyper thermal stability of α-amylases

Irreversible thermal stability of 2DOG resistant Koji mutants’ α-amylases was determined by incubating the enzyme solutions at 55 °C. Aliquots were withdrawn at regular time intervals and cooled in ice for about 30 min for the recovery. The time course aliquots were assayed for α-amylase residual activity and data was analyzed by applying pseudo first order plots [50]. The half-life was calculated by using the formula:

$$ \mathrm{Half}-\mathrm{life}\ \left(\mathrm{t}\frac{1}{2}\right)=\mathrm{ln}2/{K}_{\mathrm{d}} $$

The 2DOG resistant Koji mutants having highest α-amylase productivity, specific activity and thermal stability were finally selected.

### FESEM of Koji (*A. oryzae*)

One ml of inoculum was taken and centrifuged at 13,000 rpm for 3 min. The supernatant was discarded. The mycelia were then coated with carbon and loaded on stub to be viewed under scanning electron microscope JEOL JSM7500F.

### Aflatoxins analysis using LCMS

The cell free supernatant (two hundred ml) produced by mutant Koji strain M-100(6) was extracted (using separating funnel) with two hundred ml of chloroform. The organic solvent (chloroform) was evaporated using rotary evaporator. The residue was dissolved in methanol (two ml), filtered through membrane filter (0.45 μm, nylon membrane, Millipore) and subjected to aflatoxins analysis using LC-MS (Quadrupole Linear Ion Trap Mass Spectrometer Finnegan LTQ XL hyphenated with Surveyor Plus LC system, manufactured by Thermo Fisher Scientific, USA. Samples were analyzed using previously developed analytical method [51]. The samples were initially infused to the Mass Spectrometer by direct syringe pump through positively ionized electro spray ionization (ESI) probe: spray voltage 5 kV, sheath gas flow rate 70 arbitrary units, auxiliary gas flow rate 20 arbitrary unit, capillary temperature 335 °C, capillary voltage 45 V, and tube lens voltage 110 V.

### LC-MS/MS Analysis

Mixture of aflatoxins (aflatoxin B_1_, aflatoxin B_2_, aflatoxin G_1_ and aflatoxin G_2_) standards, having 10ng ml^-1^ concentration (each) in acetonitrile, was subjected to auto sampler (10 μl sample injection) and chromatographed on Surveyor Plus LC system (Thermo Fisher scientific) equipped with Luna® C_18_ column (150 x 4.6 mm; 3μm particle size, Phenomenex, USA), for the separation of aflatoxins analytes from samples and matrix matched external standards with an isocratic flow rate of 0.4 ml min^-1^. The column temperature was maintained at 30 °C. The injection volume was set 10 μl on auto-sampler and a mobile phase consisting of methanol:acetonitrile:water (22:22:56 *v/v*) was used for a 20 min run.

### Production of alpha amylase in bioreactors

Enzymes were produced in 10L bioreactor. The inoculums density used was 0.3g cells dl^-1^. The pH and temperature was 6.5 and 30 °C. Time course aliquots were withdrawn and analyzed for total wet cell mass, α-amylases and extracellular proteins [22].

## Additional file

Additional file 1: **Table S1.** Is the data about the clearing zone Index for the production of α-amylases by *A. oryzae* cmc1 mutants on agar plates containing starch (2.0%, *w/v*) & 0.2% Triton X100. This table contains the data of all the 52 mutants obtained by gamma rays mutagenesis. **Table S2.** Is data about the estimation of wet cell mass density in inoculum of 2-deoxy D-Glucose resistant mutant derivatives of *Aspergillus oryzae* cmc1 for α-amylase production on submerged growth conditions. (DOCX 25 kb)

## Abbreviations

2DOG: 2-deoxy-D-glucose
BGL: β-glucosidase
CFU: Colony forming unit
CI: Clearing zones index
ESI: Electro spray ionization
FESEM: Field emission scanning electron microscopy
GRAS: Generally recognized as safe
Koji: *A. oryzae* cmc1
LC-MS: Liquid chromatography-mass spectrometry
LFGM: Liquid fungal growth medium
NIAB: Nuclear institute of agriculture and biology
QRM: Quenched reaction mixture
SFGM: Solid fungal growth medium
t½: Half-life
