# Supplementary material for: Random mutagenesis of super Koji (Aspergillus oryzae): improvement in production and thermal stability of α-amylases for maltose syrup production
Source: BMC Microbiol. 2018 Nov 28;18:200. doi: 10.1186/s12866-018-1345-y (PMC6264608; doi:10.1186/s12866-018-1345-y)
Supplement: Supplementary file 1 — Table S1. Is the data about the clearing zone Index for the production of α-amylases by A. oryzae cmc1 mutants on agar plates containing starch (2.0%, w/v) & 0.2% Triton X100. This table contains the data of all the 52 mutants obtained by gamma rays mutagenesis. Table S2. Is data about the estimation of wet cell mass density in inoculum of 2-deoxy D-Glucose resistant mutant derivatives of Aspergillus oryzae cmc1 for α-amylase production on submerged growth conditions. (DOCX 25 kb) [file 12866_2018_1345_MOESM1_ESM.docx]

**Supplementry Data**

**Table S1: Clearing zone Index for the production of α-amylases by *A. oryzae* cmc1 mutants on agar plates containing starch (2.0%, w/v) & 0.2% Triton X100**

| **S. No** | **Dose** | **Mutant no.** | **Halo zone + Colony diameter (cm)** | **Colony diameter**  **(cm)** | **Clearing zone index** |
| --- | --- | --- | --- | --- | --- |
| 1 | Parent Koji |  | 2.75±0.15 | 0.85±0.051 | 3.23 |
| 2 | 0.6 kGy | 1 | 4.20±0.24 | 1.2±0.071 | 3.5 |
| 3 |  | 2 | 4.25±0.25 | 1.2±0.07 | 3.54 |
| 4 |  | 3 | 4.55±0.26 | 1.55±0.094 | 2.93 |
| 5 |  | 4 | 4.45±0.26 | 1.2±0.07 | 3.70 |
| 6 |  | 5 | 4.50±0.26 | 1.27±0.08 | 4.31 |
| 7 |  | 6 | 4.22±0.24 | 1.2±0.07 | 3.52 |
| 8 |  | 7 | 4.50±0.26 | 1.05±0.06 | 4.28 |
| 9 |  | 8 | 3.95±0.23 | 1.15±0.071 | 3.43 |
| 10 |  | 9 | 4.35±0.25 | 1.25±0.076 | 3.48 |
| 11 |  | 10 | 3.95±0.22 | 1.05±0.064 | 3.76 |
| 12 |  | 11 | 2.87±0.17 | 0.55±0.034 | 5.22 |
| 13 |  | 12 | 4.30±0.24 | 1.25±0.07 | 3.44 |
| 14 | 0.8 kGy | 1 | 4.65±0.27 | 1.5±0.09 | 3.10 |
| 15 |  | 2 | 4.80±0.28 | 1.35±0.08 | 3.55 |
| 16 |  | 3 | 3.60±0.21 | 0.95±0.057 | 3.78 |
| 17 |  | 4 | 5.20±0.3 | 1.95±0.12 | 2.66 |
| 18 |  | 5 | 4.45±0.25 | 1.25±0.07 | 3.56 |
| 19 |  | 6 | 4.45±0.26 | 1.1±0.06 | 4.04 |
| 20 |  | 7 | 4.70±0.28 | 1.35±0.08 | 3.48 |
| 21 |  | 8 | 4.75±0.27 | 1.9±0.11 | 2.50 |
| 22 |  | 9 | 4.60±0.26 | 1.2±0.07 | 3.83 |
| 23 |  | 10 | 5.85±0.34 | 1.35±0.08 | 4.33 |
| 24 |  | 11 | 5.80±0.33 | 1.25±0.07 | 4.64 |
| 25 |  | 12 | 5.90±0.34 | 1.45±0.089 | 4.06 |
| 26 | 1.0 kGy | 1 | 3.20±0.18 | 1±0.06 | 3.2 |
| 27 |  | 2 | 3.20±0.18 | 0.7±0.04 | 4.57 |
| 28 |  | 3 | 3.45±0.20 | 0.8±0.04 | 4.31 |
| 29 |  | 4 | 3.30±0.19 | 0.7±0.04 | 4.71 |
| 30 |  | 5 | 3.10±0.17 | 0.85±0.051 | 3.65 |
| 31 |  | 6 | 6.15±0.35 | 1±0.06 | 6.15 |
| 32 |  | 7 | 3.50±0.2 | 0.75±0.04 | 4.67 |
| 33 |  | 8 | 3.00±0.17 | 0.9±0.054 | 3.33 |
| 34 |  | 9 | 2.80±0.16 | 0.65±0.03 | 4.30 |
| 35 |  | 10 | 3.45±0.2 | 1.75±0.1 | 1.97 |
| 36 |  | 11 | 3.15±0.18 | 0.75±0.04 | 4.20 |
| 37 |  | 12 | 4.1±0.23 | 0.7±0.04 | 5.86 |
| 38 | 1.2 kGy | 2 | 2.10±0.12 | 0.8±0.05 | 2.62 |
| 39 |  | 3 | 2.00±0.11 | 0.7±0.04 | 2.85 |
| 40 |  | 4 | 3.10±0.17 | 0.7±0.04 | 4.43 |
| 41 |  | 5 | 4.55±0.26 | 0.92±0.056 | 4.92 |
| 42 |  | 6 | 2.50±0.14 | 0.75±0.045 | 3.33 |
| 43 |  | 7 | 4.15±0.24 | 1.9±0.11 | 2.18 |
| 44 |  | 8 | 0.95±0.05 | 0.5±0.03 | 1.90 |
| 45 |  | 9 | 2.95±0.17 | 0.85±0.051 | 3.47 |
| 46 |  | 10 | 2.80±0.16 | 0.8±0.048 | 3.50 |
| 47 |  | 11 | 4.05±0.23 | 0.7±0.04 | 5.78 |
| 48 |  | 12 | 2.75±0.15 | 0.95±0.057 | 2.89 |
| 49 | 1.4 kGy | 1 | 2.55±0.14 | 0.8±0.048 | 3.19 |
| 50 |  | 2 | 2.10±0.12 | 0.9±0.055 | 2.33 |
| 51 |  | 3 | 1.60±0.09 | 1±0.06 | 1.60 |
| 52 |  | 5 | 1.40±0.08 | 0.9±0.051 | 1.50 |
| 53 |  | 6 | 4.65±0.27 | 1.15±0.07 | 4.04 |

Clearance zone index (CI) = (halo zone diameter + colony diameter)/colony diameter. Data presented are average values ± SD of n = 3 experiments.

**Table S2: Estimation of wet cell mass density in inoculum of 2-deoxy D-Glucose resistant mutant derivatives of *Aspergillus oryzae* cmc1 for α-amylase production on submerged growth conditions**

| **S. No** | **γ-ray Exposure** | **Wet cells density**  **(g/ml)** | **Inoculum’s vol. used for α-amylase production (ml)** |
| --- | --- | --- | --- |
| 1 | Parental strain | 0.0486±0.0027 | 6.00±0.33 |
| 2 | M-60(M5) | 0.0467±0.0025 | 6.24±0.34 |
| 3 | M-80(10) | 0.1246±0.0068 | 2.34±0.13 |
| 4 | M-80(11) | 0.1310±0.0072 | 2.22±0.12 |
| 5 | M-80(12) | 0.0354±0.0019 | 8.23±0.45 |
| 6 | M-100(6) | 0.0182±0.0010 | 16.0±0.88 |
| 7 | M-100(12) | 0.0262±0.0014 | 11.14±0.61 |
| 8 | M-120(5) | 0.2507±0.0130 | 1.16±0.06 |
| 9 | M-120(11) | 0.1730±0.0095 | 1.68±0.09 |
| 10 | M-140(6) | 0.2679±0.0140 | 1.09±0.06 |

Data presented are average values ± SD of n = 3 experiments.
